# Population-Attributable Causes of Cancer in Korea: Obesity and Physical Inactivity

**DOI:** 10.1371/journal.pone.0090871

**Published:** 2014-04-10

**Authors:** Sohee Park, Yeonju Kim, Hai-Rim Shin, Boram Lee, Aesun Shin, Kyu-Won Jung, Sun Ha Jee, Dong Hyun Kim, Young Ho Yun, Sue Kyung Park, Mathieu Boniol, Paolo Boffetta

**Affiliations:** 1 Division of Cancer Registration and Surveillance, National Cancer Center, Goyang, Korea; 2 Division of Cancer Early Detection, National Cancer Center, Goyang, Korea; 3 Western Pacific Regional Office, World Health Organization, Manila, the Philippines; 4 Department of Epidemiology and Health Promotion, Institute for Health Promotion, Graduate School of Public Health, Yonsei University, Seoul, Korea; 5 Department of Social and Preventive Medicine, Hallym University, Chuncheon, Korea; 6 College of Medicine, Korea University, Seoul, Korea; 7 Department of Preventive Medicine, College of Medicine, Korea University, Seoul, Korea; 8 International Prevention Research Institute, Lyon, France; 9 The Tisch Cancer Institute, Mount Sinai School of Medicine, New York, New York, United States of America; Indiana University Richard M. Fairbanks School of Public Health, United States of America

## Abstract

**Background:**

Changes in lifestyle including obesity epidemic and reduced physical activity influenced greatly to increase the cancer burden in Korea. The purpose of the current study was to perform a systematic assessment of cancers attributable to obesity and physical inactivity in Korea.

**Methodology/Principal Findings:**

Gender- and cancer site-specific population-attributable fractions (PAF) were estimated using the prevalence of overweight and obesity in 1992–1995 from a large-scale prospective cohort study, the prevalence of low physical activity in 1989 from a Korean National Health Examination Survey, and pooled relative risk estimates from Korean epidemiological studies. The overall PAF was then estimated using 2009 national cancer incidence data from the Korea Central Cancer Registry.

Excess body weight was responsible for 1,444 (1.5%) and 2,004 (2.2%) cancer cases among men and women, respectively, in 2009 in Korea. Among men, 6.8% of colorectal, 2.9% of pancreatic, and 16.0% of kidney cancer was attributable to excess body weight. In women, 6.6% of colorectal, 3.9% of pancreatic, 18.7% of kidney, 8.2% of postmenopausal breast, and 32.7% of endometrial cancer was attributable to excess body weight. Low leisure-time physical activity accounted for 8.8% of breast cancer, whereas the PAF for overall cancer was low (0.1% in men, 1.4% in women). Projections suggest that cancers attributable to obesity will increase by 40% in men and 16% in women by 2020.

**Conclusions/Significance:**

With a significantly increasing overweight and physically inactive population, and increasing incidence of breast and colorectal cancers, Korea faces a large cancer burden attributable to these risk factors. Had the obese population of Korea remained stable, a large portion of obesity-related cancers could have been avoided. Efficient cancer prevention programs that aim to reduce obesity- and physical inactivity-related health problems are essential in Korea.

## Introduction

Over the past several decades, industrialization and urbanization has led to major lifestyle changes in developed and developing countries. These changes have resulted in steadily increasing overweight and obese populations, creating significant clinical and public health burdens worldwide. An estimated 23.2% of the world's adult population (937 million people) was overweight and 9.8% was obese in 2005 [1]. Beyond its effect on overall mortality and cardiovascular disease, excess body weight has been shown by prospective cohort studies and mortality data to contribute to an increased risk of several cancers [2], [3]. According to the International Agency for Research on Cancer (IARC), these cancers include postmenopausal breast, colorectal, uterine corpus, kidney, and esophageal adenocarcinomas [2]. Pancreatic cancer was also listed by the World Cancer Research Fund (WCRF) as a cancer site with a “convincing” association with excess body weight, and gallbladder cancer was reported to have a “probable” association [3]. Associations between obesity and cancers of the prostate, stomach, and premenopausal breast have not yet been established [3], [4]; lung cancer and esophageal squamous cell carcinoma have been suggested to have an inverse relationship with excess body weight, but conclusive evidence is currently lacking [5].

Physical inactivity is also closely linked to overweight and obesity and independently affects the risks of colon and breast cancers [2]. The lower rates of cancer among physically active persons may be due to the alteration of body fat or sex hormone levels, intestinal transit time, and/or immune function [6].

The prevalence of overweight and obesity has increased markedly in Western developed countries since the 1970s. Rapid industrialization and urbanization since the 1980s have affected the global population, including Asia-Pacific countries [7]–[9]. Humans have become more sedentary as mechanization has eliminated much of the need for physical activity, which has resulted in increased obesity. Although many epidemiological studies have examined the association between body weight and cancer incidence or mortality, almost all of the relevant data have been collected from Western populations. Asians tend to have smaller body frames than Caucasians, and their lifestyles, genetic backgrounds, and general environments also differ. One report has demonstrated a U-shaped relationship between body weight and mortality in Asian populations, unlike Caucasian populations [8]. The application of the World Health Organization's (WHO) standard classification of obesity (body mass index (BMI)≥30 kg/m^2^) to Asian populations or other ethnic groups has also been debated, as it was developed with data from Western populations. Asians tend to have a lower average BMI and a higher percentage of body fat at any given BMI [10]. The WHO Western Pacific Region and other organizations formed a task force that suggested a different obesity standard (BMI≥25 kg/m^2^) for Asian populations [8]. Thus, the results of overweight and disease risk studies in Asia should be interpreted with caution, and reliable evidence from Asian populations is essential.

With lifestyle changes due to industrialization and urbanization, Korea faces a serious health problem related to obesity. The prevalence of obese adults (BMI≥25 kg/m^2^) in Korea was 30.6% (32.4% in men, 29.4% in women) in 2001, representing a large increase since the 1990s [9]. Korea also has the highest cancer incidence and mortality rates among Asian countries, where cancer is the leading cause of death [11]. In 2009, there were 325.5 and 259.3 newly diagnosed cancer cases per 100,000 men and women, respectively, and the cancer mortality rates were 97.8 per 100,000 [12].

To measure the impact of obesity and physical inactivity and to establish population-level cancer prevention strategies, it is important to estimate the population-attributable fraction (PAF) of cancer for excess weight and physical inactivity. The obesity attributable fraction of cancer has been estimated for several Western regions, including a recent report from 30 European countries [2], [4], [5], [13], but little information on the PAF of obesity and physical inactivity in Asian countries has been published to date. The objective of the current study was to estimate the number of cancer cases in Korea that is attributable to obesity and physical inactivity. This study was conducted as part of a systematic analysis of attributable causes of cancer in Korea.

## Materials and Methods

### Definition of excess body weight (overweight, obesity) and physical inactivity

In consideration of an approximately 20-year induction period from exposure to excess weight or physical inactivity to the development of cancer, the prevalence of obesity and physical inactivity in years close to early 1990s was considered for estimating associated cancer cases in 2009. Data on the prevalence of overweight and obesity were obtained from a large-scale population-based cohort study by the National Health Insurance Corporation that gathered baseline information for more than 1,200,000 subjects in 1992–1995 [14]. Measured weight and height were used to calculate BMI (in kg/m^2^). We used the recommended BMI cut-off points for Asian populations to define “overweight” (23 kg/m^2^≤BMI<25 kg/m^2^) and “obesity” (BMI≥25 kg/m^2^) in this study [8].

Physical activity data from the 1989 Korea National Health Examination Survey (KNHES) were used. The KNHES is a representative survey designed to provide reliable nationwide statistics on health status, health-related behavior, and perceived health. Our definition of physical activity was based on the combined information from self-reported frequency and duration of recreational physical activity. Low leisure-time physical activity was defined as regularly exercising less than once per week for at least 15 minutes per session.

### Estimation of relative risks

Relative risks (RRs) for each cancer site associated with obesity and physical inactivity were evaluated using reported epidemiological studies in Korea. We conducted a comprehensive literature search for studies published in English or Korean before August 2012 in PubMed (http://www.ncbi.nlm.nih.gov/pubmed/) and KoreaMed (http://www.koreamed.org/SearchBasic.php) using the search keywords “Korea,” “obesity,” “overweight,” “excess weight,” “physical activity,” “physical inactivity,” and “cancer.” Inclusion criteria for literature search was that the study should be epidemiological studies conducted on the Korean population and provided the information to estimate the RRs or ORs for cancer incidence. Additional citations were identified from the reference lists of the resulting articles and with information provided by cancer experts in Korea. When there were multiple reports of the same study, the publication with the longest follow-up period or the largest event numbers was selected for the estimation of pooled RRs. When necessary, we obtained additional data through personal communication with the author(s) of the published articles [14]. The initial literature search identified 30 studies on obesity and cancer, but many of these were excluded from the final analysis by following exclusion criteria: risk estimates and/or precise information (e.g., standard errors, 95% confidence intervals) were not available or the classification of overweight and obesity was different (19 studies); and multiple results were reported from the same study population (4 studies). Finally seven studies on obesity were included in the meta-analysis [14]–[20]. For physical activity, seven studies were initially identified, four of which were excluded because the classification of physical activity was rather different (for example, only defined as “yes” and “no”), and three studies [16], [21], [22] were used for the final evaluation of RRs (Tables S1, S2). Out of nine studies used for excess body weight and physical activity, two studies were cohort studies and seven studies were case-control studies. When the outcome is a rare event, RRs can be estimated by ORs. As we dealt with cancer incidences that are very rare, we did not separate the estimates from cohort studies and those from case-control studies when performing meta-analyses. Because we found no reported examination of the association between colorectal cancer and physical activity in Korean women, we used the estimated risk value obtained from men.

Meta-analyses were performed to estimate the pooled RRs and 95% confidence intervals (CIs) for fixed- and random-effects models. In cases of heterogeneity identified by I^2^ and Q statistics, the risk estimates from the random-effects model were used [23]. The analyses were performed using the “Metan” command in the Stata program (ver. 10.0; StataCorp, College Station, TX, USA) and Comprehensive Meta-Analysis software (ver. 2; Biostat, Englewood, NJ, USA).

### Cancer incidence data

The most recent available annual cancer incidence data (2009) were obtained from The Korea Central Cancer Registry, a population-based nationwide cancer registry in Korea [12]. This review considered cancer sites that showed a convincing positive association with obesity or physical inactivity: colorectal, pancreatic, kidney, esophageal (adenocarcinomas), uterine corpus, and postmenopausal breast cancers for obesity, and colon and breast cancers for physical inactivity. As the menopausal status of breast cancer patients was not reported in the nationwide cancer incidence database, we regarded breast cancer cases in women aged 50 years or older to be postmenopausal. We did not include esophageal cancer in the PAF calculation because it showed a significant inverse relationship with obesity in men (hazard ratio = 0.55 for obese *vs.* normal weight) and no relationship with obesity in a large Korean prospective study (additional analysis of Jee et al.) [14], and it has a relatively low incidence in Korea [12].

### Estimation of population-attributable fraction (PAF)

Gender- and cancer site-specific PAFs were calculated using the following modified Levin's formula for multiple categories (k), proposed by Hanley [24], [25]:
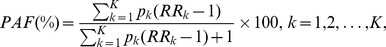
where RR is the relative risk of a risk factor for a specific cancer, P is the prevalence of exposure to the risk factor in the total population, and K is the number of categories in the risk factor. The counterfactual exposure for excess body mass was BMI<23 kg/m^2^ while excess body weight groups included two separate categories of 23≤BMI<25 kg/m^2^ (overweight category) and BMI≥25 kg/m^2^ (obese category) and that for leisure-time physical activity was light to moderate (≥1 exercise session per week and ≥15 minutes per session). When the estimated RR was less than 1 for risk factors, RR = 1 was used instead for PAF calculation.

### Sensitivity analysis and projected obesity-related PAF

Sensitivity analyses were performed for the PAF for obesity by using the lower and upper bounds of the 95% CIs for RR estimates. We also investigated the sensitivity of our PAF results using different BMI cut-off points (≥25 kg/m^2^ vs. ≥30 kg/m^2^)for the definition of obesity. The projected PAF for excess body weight in 2020 was calculated by using the projected obesity prevalence extrapolated from a linear trend of previous obesity prevalence into the future.

## Results

The prevalence of overweight (23 kg/m^2^≤BMI<25 kg/m^2^) was 28.5% among men and 23.2% among women, and the prevalence of obesity (BMI≥25 kg/m^2^) was 23.9% among men and 26.8% among women in 1992–1995. More Korean men (21.6%) than women (12.2%) reported light to moderate leisure-time physical activity in the 1989 KNHES. The RRs for obesity and related cancers are summarized in Table 1. The risks differed by gender and cancer type. Although our pooled estimates found no significant association between pancreatic cancer and excess weight, they did show significantly increased risks for colorectal, kidney, uterine corpus, and postmenopausal breast cancers in overweight and obese people. Specifically, the risks for kidney (RR 1.46 for obese men; RR 1.40 for obese women) and uterine corpus cancers (RR 2.65 for obese women) were high. Compared with light to moderateleisure-time physical activity, low physical activity increased the risk of breast cancer by 10%, but was not associated with colorectal cancer.

**Table 1:** Relative risk for excess body weight and physical inactivity and related cancers in Korea (using Asian and Caucasian cut-offs for body mass index [BMI]).

Cancer site(ICD-10 code)	Men		Women	
	Excess body weight (BMI*, kg/m^2^)			
	23≤BMI<25	BMI≥25	23≤BMI<25	BMI≥25
Colorectum (C18-C20)**	1.12 (1.06–1.19)	1.16 (1.10–1.24)	1.12 (1.06–1.19)	1.16 (1.10–1.24)
Pancreas (C25)	1.03 (0.92–1.15)	1.09 (0.97–1.22)	0.93 (0.77–1.12)	1.15 (0.98–1.35)
Kidney (C64)	1.28 (1.13–1.46)	1.46 (1.28–1.66)	1.53 (1.17–2.01)	1.40 (1.07–1.83)
Breast (C50)^c^	-	-	0.92 (0.77–1.11)	1.26 (1.05–1.51)
Uterine corpus (C54)	-	-	1.19 (0.62–2.29)	2.65 (1.44–4.89)

	25≤BMI<30	BMI≥30	25≤BMI<30	BMI≥30
Colorectum (C18-C20)**	1.08 (0.99–1.16)	1.42 (1.02–1.98)	0.92 (0.79–1.07)	1.01 (0.72–1.42)
Pancreas (C25)	1.06 (0.90–1.24)	1.34 (0.75–2.38)	1.35 (1.05–1.74)	1.80 (1.14–2.86)
Kidney (C64)	1.11 (0.93–1.31)	1.38 (0.76–2.52)	0.92 (0.64–1.31)	1.21 (0.58–2.53)
Breast (C50)***	-	-	1.07 (0.91–1.24)	1.54 (1.11–2.13)
Uterine corpus (C54)	-	-	1.14 (1.03–1.27)	1.25 (1.04–1.50)

Cancer site(ICD-10 code)	Physical inactivity (low vs. light-moderate physical activity)		
Breast (C50)	Low	-	1.01 (0.92–1.10)
Colon (C18)	Low	1.01 (0.92–1.10)	1.11 (0.35–3.49)

* BMI, body mass index, *kg/m^2^*.

** Relative risk for colon cancer was estimated for men and women combined and also used for rectal cancer.

*** Breast cancer in postmenopausal women.

Table 2 presents the overall, gender-, and cancer site-specific PAFs of cancer cases attributable to obesity and physical inactivity. The overall and obesity-related PAFs were higher among women than among men. Excess body weight was responsible for 1,444 (1.5%) and 2,004 (2.2%) cancer cases among Korean men and women in 2009, respectively. While the overall PAF attributable to excess overweight or physical inactivity was relatively low, cancer site-specific PAFs were relatively high. For example, the PAF of excess body weight for kidney cancer in both men and women (16.0% in men, 18.7% in women) was substantial. Among women, 6.6% of colorectal, 3.9% of pancreatic, 8.2% of postmenopausal breast and 32.7% of uterine corpus cancer were attributable to excess body weight. Among men, 6.8% of colorectal, and 2.9% of pancreatic cancer were attributable to excess body weight.

**Table 2:** Cancer cases attributable to obesity and physical inactivity in Korea (2009).

Obesity						
Cancer site	Men			Women		
	PAF^a^	No. of cases	Obesity-related Cases	PAF	No. of cases	Obesity-related cases
Colorectum	6.8	14,949	1,010	6.6	9,776	646
Pancreas	2.9	2,356	69	3.9	1,901	73
Kidney	16.0	2,290	365	18.7	1,068	200
Breast (postmenopausal)	-		-	8.2	6,331	520
Uterine Corpus	-		-	32.7	1,728	565
Total	**1.5**	96,826	1,444	**2.2**	91,068	2,004

Leisure-time physical Inactivity^b^						
Cancer site	Men			Women		
	PAF	No. of cases	Physical inactivity-related cases	PAF	No. of cases	Physical inactivity-related cases
Colorectum	0.8	14,949	117	0.9	9,776	85
Breast	-	-	-	8.8	13,352	1,176
Total	**0.1**	96,826	117	**1.4**	91,068	1,261

a PAF, population-attributable fraction.

b Prevalence of low leisure-time physical activity obtained from the 1989 Korean National Health Examination Survey (78.4% men, 87.8% women).

With regard to physical inactivity, 0.8% of colorectal cancers in men and 0.9% in women were attributable to low physical activity. The PAF for physical inactivity was very small for men. Physical inactivity accounted for 8.8% of female breast cancer in Korea.

Table 3 shows the past and projected prevalences of overweight and obesity in Korea. The number of Koreans with excess body weight has increased steadily in the last 15 years; based on these data, the proportion of obese (BMI≥25 kg/m^2^) men is forecast to almost double (from 23.9% to 46.3%), and that for women are expected to increase from 26.8% to 33.1% by 2020. Although fewer Koreans than Westerners are overtly obese (BMI≥30 kg/m^2^), the proportion of Koreans in this category is also rapidly increasing.

**Table 3:** Prevalence (%) of excess body weight in the Republic of Korea.

Year	Men			Women		
	BMI<23	23≤BMI<25	BMI≥25	BMI<23	23≤BMI<25	BMI≥25
**1992–1995** *	**47.6**	**28.5**	**23.9**	**50.0 (39.2)**	**23.2 (26.3)**	**26.8 (34.4)**
1998**	50.2	24.4	25.4	50.5 (38.8)	21.3 (24.0)	28.2 (37.2)
2005**	36.2	27.4	36.4	46.9 (33.5)	22.9 (26.0)	30.2 (40.5)
2007**	38.1	27.1	34.8	46.8 (31.7)	22.5 (25.8)	30.7 (42.5)
2009**	39.2	25.2	35.6	48.5 (35.5)	21.7 (26.0)	29.8 (38.5)
2015***	32.4	25.6	42.0	46.1 (30.7)	22.0 (25.9)	32.0 (43.4)
2020***	28.5	25.2	46.3	45.1 (28.6)	21.8 (26.1)	33.1 (45.3)

Year	Men			Women		
	BMI<25	25≤BMI<30	BMI≥30	BMI<25	25≤BMI<30	BMI≥30
1996–1997*	72.52	26.13	1.35	69.31	27.54	3.15
**1998** **	**74.6**	**23.74**	**1.67**	**71.81 (62.8)**	**24.98 (33.1)**	**3.21 (4.1)**
2005**	63.61	33.38	3.01	69.82 (59.5)	26.58 (36.4)	3.60 (4.1)
2015***	52.74	42.39	4.87	69.82 (49.7)	26.07 (45.5)	4.11 (4.9)

- Prevalence in postmenopausal is in parenthesis.

- BMI categories (kg/m^2^) for Asians: reference group, <23.0; overweight, 23.0–24.9.0; obese, ≥25.0;

- BMI categories (kg/m^2^) for Caucasians: reference group, <25.0; overweight, 25.0–29.0; obese, ≥30.0.

* National Health Insurance Corporation data in 1992–1995.

** Korea National Health and Nutrition Examination Survey.

*** Prevalence projected from Korean National Health and Nutrition Examination Survey data in 1992–2009.

Table 4 shows the changes in projected PAFs using the overweight and obesity prevalences at different time points. The PAFs of overweight- and obesity-related cancer (colorectum, pancreas and kidney) are expected to increase from 1.5% to 2.1% for men and from 2.2% to 2.6% for women (colorectum, pancreas, kidney, breast and uterine corpus), with gradual increases observed between periods. This implies that an additional 586 (40% increase) male cancer cases and 322 (16% increase) female cancer cases will be attributable to overweight and obesity in 2020 compared with the early 1990s and assuming the same level of cancer incidence as in 2009.

**Table 4:** Projected population-attributable fraction for excess body weight in Korea.

Year of obesity prevalence		1992 – 1995		1998		2005		2015*		2020*		2020 vs. 1992 - 1995
Cancer site	No. of Cases	PAF(%)	No. of attributable cases	PAF(%)	No. of attributable cases	PAF(%)	No. of attributable cases	PAF(%)	No. of attributable cases	PAF(%)	No. of attributable cases	Difference in obesity-related cases
**Men**												
Colorectum	14,949	6.8	1,010	6.5	977	8.4	1,248	8.9	1,333	9.5	1,412	403
Pancreas	2,356	2.9	69	2.9	69	3.9	93	4.4	102	4.7	111	42
Kidney	2,290	16.0	365	15.6	358	19.6	449	20.9	480	22.1	506	141
Total	96,826		1,444		1,404		1,790		1,915		2,029	586
% of all cancers			1.5		1.5		1.9		2.0		2.1	
**Women**												
Colorectum	9,776	6.6	645	6.6	645	7.1	689	7.2	704	7.3	717	71
Pancreas	1,901	3.9	73	4.1	77	4.3	82	4.6	87	4.7	90	17
Kidney	1,068	18.7	200	18.4	197	19.5	208	19.7	210	19.9	212	12
Breast	6,331	8.2	520	8.8	558	9.5	603	10.1	642	10.5	667	148
Corpus uteri	1,728	32.7	565	33.6	580	35.1	607	36.3	627	37.0	640	74
Total	91,068		2,004		2,058		2,190		2,270		2,326	322
% of all cancers			2.2		2.3		2.4		2.5		2.6	

Notes: 1) Obese and overweight prevalence data were estimated from the large-scale cohort study of National Health Insurance Corporation in1992-1995, from Korea National Health and Nutrition Survey in 1998 and 2005, and from extrapolating to 2015 and 2020; cancer incidence in 2009 was used for the calculation of projected PAF and obesity-related cases.

2) BMI categories (kg/m^2^): reference group, <23.0; overweight, 23.0–24.9; obese, ≥25.

* Using projected prevalence of obesity in 2015, 2020 as shown in Table 3.

**Figure 1:**
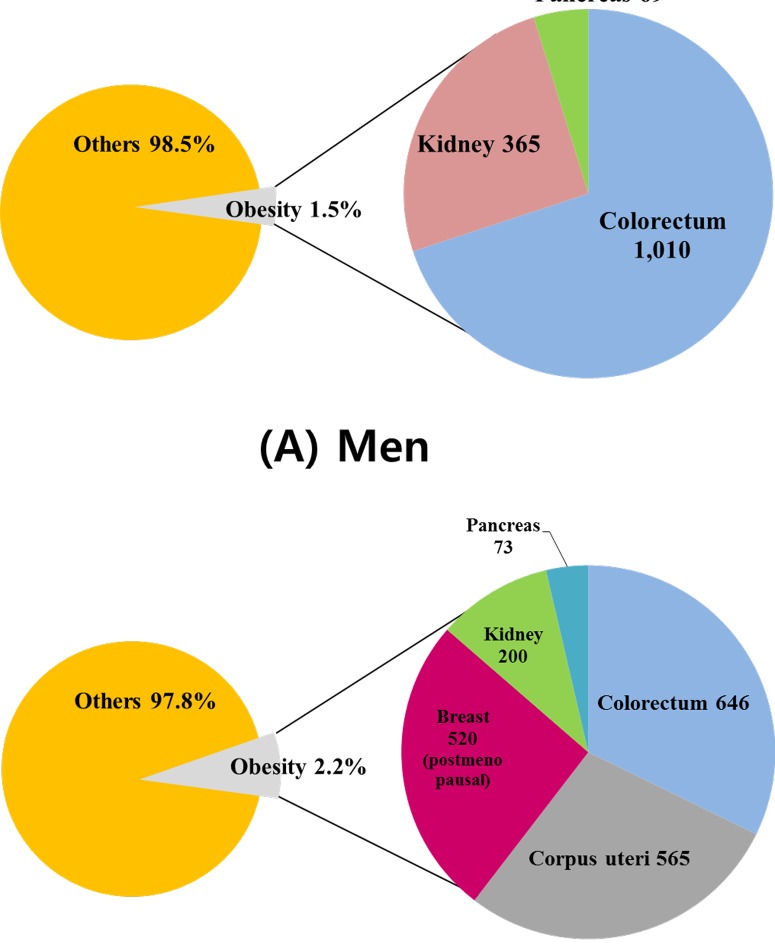
Population-attributable fractions for obesity by cancer site: (A) for Korean men and (B) for Korean women.

**Figure 2:**
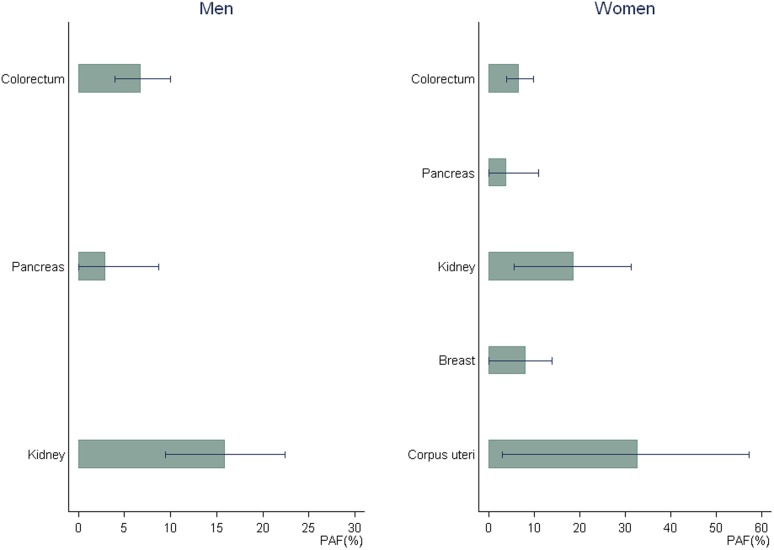
Sensitivity analysis on the estimation of population-attributable fractions for obesity using the lower and upper bounds of 95% confidence intervals of relative risks.

A visual representation of the cases attributable to overweight and obesity by cancer site shows that colorectal cancer made up over half of all obesity-related cancer cases in men (Figure 1A). In women, breast and uterine corpus cancers accounted for more than half of all obesity-related cancer cases (Figure 1B). Sensitivity analysis showed that the PAF estimates were more sensitive to RR variation in women than in men, most likely due to the larger degree of uncertainty in the estimation of RRs for women (Figure 2). In our study, uterine corpus cancer had the widest range of estimated PAF due to the degree of uncertainty in the RR.

## Discussion

Our study systematically assessed cancer incidence and mortality attributable to excess body weight and physical inactivity in Korea. The overall PAFs for cancer incidence related to excess weight were 1.5% among men and 2.2% among women. Our overall PAF for excess body weight among Koreans was slightly higher than Japan and lower than the United States and UK (Table 5) [26], [27]. Several factors may explain the differences in the overall PAFs of Korea and the United States or UK. Overweight is likely less prevalent in Korea than in the United States or UK. However, our study used the Asia-Pacific standards for excess weight (BMI≥23 kg/m^2^) to obtain overweight prevalences of 62.4% for men and 50.0% for women. The American study used the overtly obese standard (BMI≥30 kg/m^2^) to obtain prevalences of 33.3% for men and 35.3% for women from the 2005–2006 National Health and Nutrition Examination Survey (NHANES) data, and the UK study used prevalences of overweight and obese population by age groups in 2000–2001, using data from the Nation Diet and Nutrition Survey (19–64 years) and Health Survey for England (>65 years) [27], [28]. When overtly obese standard (BMI≥30 kg/m^2^) was used for the Korean data, the discrepancy between the countries' PAFs was even larger (Table S3).

**Table 5:** International comparison of population-attributable fraction for excess body weight on cancer.

Cancer site (ICD-10)	Korea						Japan [26]				United States [28]				United Kingdom [27]			
	Men			Women			Men		Women		Men		Women				Men	Women
	RR		PAF	RR		PAF	RR	PAF	RR	PAF	RR	PAF	RR	PAF	RR		PAF	PAF
	23≤BMI <25	BMI≥25		23≤BMI <25	BMI≥25		BMI≥25		BMI≥25		BMI≥30		BMI≥30		25≤BMI <30	BMI≥30		
Colorectum (C18-20)	1.12	1.16	6.8	1.12	1.16	6.6					1.4	12	1.1	3	1.15	1.32	13.6	12.2
Colon (C18)							1.24	5.2	1.17	4.0								
Pancreas (C25)	1.03	1.09	2.9	0.93	1.15	3.9	0.7	-	1.1	2.4	1.2	6	1.2	7	1.14	1.3	12.8	11.5
Breast (C50) (postmenopausal; aged 50 and more)	-	-	-	0.92	1.26	8.2	-	-	1.12	2.9	-	-	1.3	10	1.12	1.25	-	9.0
Corpus uteri(C54)	-	-	-	1.19	2.65	32.7	-	-	1.73	15.3	-	-	-	-	1.52	2.31	-	33.7
Kidney (C64)	1.28	1.46	16.0	1.53	1.40	18.7	1.66	13.2	1.55	12.0	2.0	25	2.0	26	1.31	1.72	25.0	22.2
Esophagus (C15) (adenocarcinoma)											3.0	40	2.6	36	1.55	2.4	26.9	11.2
Gallbladder (C23)											1.4	12	2.0	26	1.23	1.51	19.7	17.8
Prevalence (%)																		
Overweight	26.1 (28.5)*			27.5 (23.2)*			23.0**		24.7**		-		-			25∼52†	25∼41†	
Obesity	1.4 (23.9)*			3.2 (26.8)*							33.3		35.3			17∼32†	14∼30†	
% of all cancers			1.5			2.2		0.8		1.6		4.4€		7.5€		4.1	6.9	

* Prevalence categorized for 25≤BMI<30, BMI≥30 from the 1996–1997 records of the National Health Insurance Cooperation; parenthesis represent each of 23≤BMI<25, BMI≥25 from 1992–1995 records of the National Health Insurance Cooperation, which were used for PAF calculation.

** Prevalence data for 1990 from pooled analysis of 6 prospective cohort studies, categorized BMI≥25 for overweight and obesity.

† Range of prevalence by age groups in 2000–2001, data from the Nation Diet and Nutrition Survey (ages 19–64)/Health Survey for England (ages >65) [27].

€ PAF also includes contribution from cancers of endometrium, gastric cardia, liver, thyroid, and leukemia, myeloma, and non-Hodgkin's lymphoma.

The PAF differences also appear to derive from higher RRs in the American population than in Korean or European populations. Polednak [28] has noted that the American PAF may have been overestimated because the RRs in that study were obtained from comparison of an obese group to a “normal” BMI group, usually defined as <25 kg/m^2^, 18.5–24.9 kg/m^2^, or 22.5–24.9 kg/m^2^, rather than a completely non-obese group. Furthermore, the total PAF estimation in the United States and UK included other cancer types, such as cancers of endometrium, gastric cardia, liver, thyroid, leukemia, myeloma, and non-Hodgkin's lymphoma, whereas our study included only cancer sites convincingly associated with obesity. Differences in the distribution of cancer incidence may also be a source of variation in the overall PAFs among countries.

Site-specific PAFs were higher in the American population than among Koreans for all cancer sites except female colorectal cancer. The PAFs of colorectal (6.8%) among men in our study was about half of that in the UK. The PAF for postmenopausal breast cancer in the present study (8.2%) was higher than Japan (2.9%) and very slightly lower than the United States (10%) and UK (9.0%) While our study did not evaluate the PAF for esophageal adenocarcinoma (see materials and methods section), it was very high in European and American populations (Table 5). Our estimate for pancreatic cancer (2.9% in men, 3.9% in women) was lower than those for UK (12.8% in men, 11.5% in women) and Americans (6% in men, 7% in women), reflecting previous findings that obesity increases the risk of pancreatic cancer among men and women in North American and among women in Europe and Australia women, but not in other areas [5].

The present study obtained conservative estimates for cancers attributable to low leisure-time physical activity: only 0.1% and 1.4% of total cancer incidence in men and women, respectively. We considered only the two cancer sites (breast and colorectum) that are known to be convincingly associated with physical inactivity. The RR for colorectal cancer in Korea is very low.

Our study used the definitions of overweight and obesity recommended for Asian populations by the WHO (non-overweight = BMI<23.0 kg/m^2^, overweight = BMI 23.0–24.9 kg/m^2^, obese = BMI≥25.0 kg/m^2^) [8]. We also investigated the impact on PAF estimation of using the standard BMI cut-offs (non-overweight = BMI<25.0 kg/m^2^, overweight = BMI 25.0–29.9 kg/m^2^, obese = BMI≥30.0 kg/m^2^). The PAF estimates for excess body weight were smaller when using the standard cut-offs (Table S1), which indicates the importance of employing Asian cut-offs for BMI when burden due to obesity is considered in Korea.

Korea faces a serious health problem related to obesity and cancer. The future incidence of obesity-attributable cancer will be influenced by trends in obesity prevalence and the aging of the population. As illustrated in Table 3, overweight and obesity have increased steadily among men and women in Korea, and obesity is projected to be highly prevalent by 2015 if this trend continues without intervention. Approximately 2% of the cancer burden in Korea was due to obesity 15 to 20 years ago, which may appear minimal compared with other risk factors, such as smoking. However, this figure reflects the past situation; as lifestyle changes occur, the proportion of obesity-related cancer will increase rapidly. This trend is apparent in the projected PAF estimate. The projected obesity-related cancer burden shown in Table 4 is a conservative estimate based on the 2009 cancer incidence in Korea. This conservative figure suggests that even without an increase in the obese population, at least 40% of obesity-related cancers in men and 16% in women could be avoided.

Korea leads the Asian region in cancer incidence and mortality rates [11]. Cancer incidence has increased steadily in Korea, with an annual percent change (APC) of 2.3% in the last decade, and this growth is expected to continue in the future [12]. In particular, breast (6.3% APC) and colorectal (6.2% APC) cancer show significantly increasing trends that will increase the cancer burden related to obesity and physical inactivity. The population of Korea is also aging rapidly, which will also increase the cancer burden.

The present study has several strengths. First, we used national cancer incidence data that achieve a nearly complete coverage of the Korean population. With well-established nationwide population-based cancer registration system, we had access to precise numbers of gender- and site-specific cancer cases for PAF estimation. Second, our obesity and physical inactivity data were also gathered from a representative national survey, which allowed us to accurately estimate population-attributable risks for Korea. Third, we used adjusted RRs in our PAF calculation. Many previous estimations of PAF or RR for obesity have been criticized for failing to control for confounding variables, such as smoking and drinking, which would bias RR estimates (usually through overestimation) [29]. When we investigated the distribution of obesity by age, smoking and drinking in Korean men and women, there were substantial differences in smoking or drinking prevalence among obese and non-obese population (Table S4, S5). Our adjusted RRs for obesity were controlled for confounding variables including age, smoking, and drinking, and we believe this effort strengthened the validity of causal inferences and minimized bias in our estimation of PAFs.

We also recognize the following limitations of our study. First, the restriction of our RR estimation to studies performed on Korean populations limited our sample size, which may have introduced slightly increased uncertainty in the pooled estimate of RRs, and hence uncertainty in the PAFs. However, one study included in our analysis was a very large-scale population-based prospective study with over one million subjects, which we believe reduced the degree of uncertainty in our RR estimation. Second, our assessment of physical activity only considered the leisure-time physical activities and omitted occupational, transport, and household activities. We assumed an induction period of 20 years between the time of exposure and the occurrence of cancer, and the available population-level physical activity data from the early 1990s was limited to a simple questionnaire from the KNHES. Finally, our overall PAF for obesity may have been underestimated because we only included the cancer sites with convincing evidence of association. Gallbladder, thyroid, and prostate cancer, melanoma, and non-Hodgkin's lymphoma were thereby not included.

Our results are meaningful from several perspectives. This is among the first studies to systematically assess the total proportion of cancer incidence attributable to obesity and physical inactivity in an Asian country. The large discrepancy between Korea and the United States in the proportion of obesity-related cancer poses an important question and indicates potential problems in directly applying evidence from Western countries to Asian or other non-Caucasian populations. The current study indirectly suggests the importance of population-specific overweight and obesity standards for Asians, including those residing in Western countries. For instance, obesity is not considered to be a large problem among Asian populations in the United States because only a very small proportion of Asian Americans have a BMI>30 kg/m^2^ [30]. However, a greater and rapidly increasing proportion of Asian-Americans have a BMI>25 kg/m^2^. The results of this study provide important evidence that may be used in the development of cancer prevention strategies and cancer control programs in Korea and in other parts of Asia that share similar lifestyles and genetic backgrounds.

## Supporting Information

Table S1 Studies included in the meta-analysis for excess body weight.
(DOCX)

Table S2 Studies included in the meta-analysis for physical inactivity.
(DOCX)

Table S3 Estimated number of cancer incidence cases attributable to excess body weight in the Republic of Korea (using Caucasian cut-offs).
(DOCX)

Table S4 Distribution of age, obesity, smoking and drinking in Korean men.
(DOCX)

Table S5 Distribution of age, obesity, smoking and drinking in Korean women.
(DOCX)
