# Correction: Population-Attributable Causes of Cancer in Korea: Obesity and Physical Inactivity

**DOI:** 10.1371/journal.pone.0102930

**Published:** 2014-07-11

**Authors:** The PLOS ONE Staff

The image for Figure 1 is incorrect. Please see the correct Figure 1 here.

**Figure 1:**
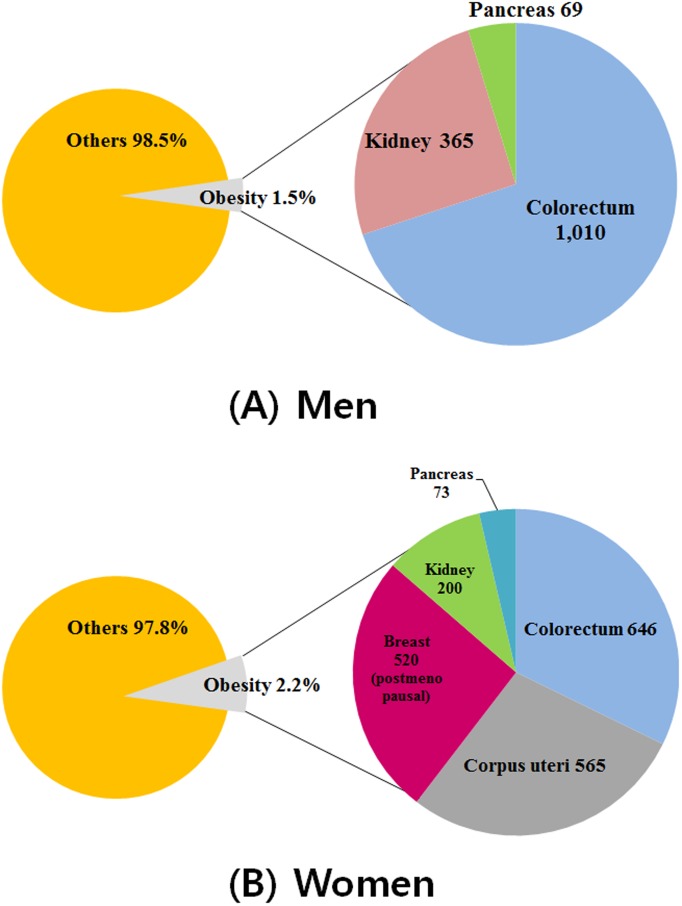
Population-attributable fractions for obesity by cancer site: (A) for Korean men and (B) for Korean women.
