# Supplementary material for: Population-Attributable Causes of Cancer in Korea: Obesity and Physical Inactivity
Source: PLoS One. 2014 Apr 10;9(4):e90871. doi: 10.1371/journal.pone.0090871 (PMC3982956; doi:10.1371/journal.pone.0090871)
Supplement: Table S1 — Studies included in the meta-analysis for excess body weight. (DOCX) [file pone.0090871.s001.docx]

Table S1. Studies included in the meta-analysis for excess body weight

| Author (year), | Study period | Study subjects | | | | Category of  BMI (kg/m^2^) | OR  (95% CI) | Confounding variables  considered |
| --- | --- | --- | --- | --- | --- | --- | --- | --- |
|  |  | Type and source | Definition | No. of  cases | No. of controls |  |  |  |
| **Colorectum** |  |  |  |  |  |  |  |  |
| Chung YW  et al. (2006) | 2002-2004 | Hanyang University Guri  Hospital | Cases; histological  Confirmed  Colorectal cancer  patients  Control; patients  of the same hospital | 37  32  36 | 48  36  21 | ~22.9  23.0~24.9  ≥25 | 1.00  1.40 (0.60-3.30)  2.30 (0.90-5.80) | Both Men and Women  Adjusted age, sex, BMI, Glucose ,TG, Cholesterol |
| Jee SH et al.  (2008) | 1992-1995 | National Health  Insurance Corporation | Cohort study | 2,756  1,915  1,909 |  | ~22.9  23.0~24.9  ≥25 | 1.00  1.12 (1.06-1.19)  1.16 (1.09-1.23) | Both Men and Women  Adjusted for age, age^2^, drinking, smoking |
|  |  |  |  | 1,415  1,218  59 |  | <25.0  25.0~29.9  ≥30.0 | 1.00  1.08 (0.99-1.19)  1.42 (1.02-1.98) | Men  Adjusted for age, smoking status |
|  |  |  |  | 509  573  68 |  | <25.0  25.0~29.9  ≥30.0 | 1.00  0.92 (0.79-1.07)  1.01 (0.72-1.42) | Women  Adjusted for age, smoking status |
| **Pancreas** |  |  |  |  |  |  |  |  |
| Jee SH et al.  (2008) | 1992-1995 | National Health Insurance Corporation | Cohort study | 873  528  463 |  | ~22.9  23.0~24.9  ≥25 | 1.00  1.03 (0.92-1.15)  1.09 (0.97-1.22 | Men  Adjusted for age, age^2^, drinking, smoking |
|  |  |  |  | 323  175  287 |  | ~22.9  23.0~24.9  ≥25 | 1.00  0.93(0.77-1.12)  1.15(0.98-1.35) | Women  Adjusted for age, age^2^, drinking, smoking |
|  |  |  |  | 524  442  17 |  | <25.0  25.0~29.9  ≥30.0 | 1.00  1.06 (0.90-1.24)  1.34 (0.75-2.38) | Men  Adjusted for age, smoking status |
|  |  |  |  | 178  253  34 |  | <25.0  25.0~29.9  ≥30.0 | 1.00  1.35 (1.05-1.74)  1.80 (1.14-2.86) | Women  Adjusted for age, smoking status |

Table S1. Studies included in the meta-analysis for excess body weight (continued)

| Author (year), | Study period | Study subjects | | | | Category of  BMI (kg/m^2^) | OR  (95% CI) | Confounding variables  considered |
| --- | --- | --- | --- | --- | --- | --- | --- | --- |
|  |  | Type and source | Definition | No. of  cases | No. of controls |  |  |  |
| **Kidney** |  |  |  |  |  |  |  |  |
| Jee SH et al.  (2008) | 1992-1995 | National Health Insurance Corporation | Cohort study | 524  424  406 |  | ~22.9  23.0~24.9  ≥25 | 1.00  1.28 (1.13-1.46)  1.46 (1.28-1.66) | Men  Adjusted for age, age^2^, drinking, smoking |
|  |  |  |  | 117  101  115 |  | ~22.9  23.0~24.9  ≥25 | 1.00  1.53(1.17-2.01)  1.40(1.07-1.83) | Women  Adjusted for age, age^2^,  drinking, smoking |
|  |  |  |  | 425  392  16 |  | <25.0  25.0~29.9  ≥30.0 | 1.00  1.11 (0.93-1.31)  1.38 (0.76-2.52) | Men  Adjusted for age, smoking  Status |
|  |  |  |  | 100  100  14 |  | <25.0  25.0~29.9  ≥30.0 | 1.00  0.92 (0.64-1.31)  1.21 (0.58-2.53) | Women  Adjusted for age, smoking status |
| **Breast** |  |  |  |  |  |  |  |  |
| Jee SH et al.  (2008) | 1992-1995 | National Health Insurance Corporation | Cohort study | 188  139  207 |  | ~22.9  23.0~24.9  ≥25 | 1.00  1.04 (0.84-1.30)  1.25 (1.02-1.53) | Postmenopausal Women  Adjusted for age, age^2^, drinking, smoking |
| Kim J et al.  (2009) | 2007-2008 | National Cancer Center Hospital | Cases: histologically confirmed breast cancer patients Controls: patients of the same hospital | 169  85  104 | 158  121  75 | <23.0  23.0~24.9  ≥25 | 1.00  0.66(0.45-0.95)  1.30(0.88-1.91) | Pooled Estimation |

Table S1. Studies included in the meta-analysis for excess body weight (continued)

| Author (year), | Study period | Study subjects | | | | Category of  BMI (kg/m^2^) | OR  (95% CI) | Confounding variables  considered |
| --- | --- | --- | --- | --- | --- | --- | --- | --- |
|  |  | Type and source | Definition | No. of  cases | No. of controls |  |  |  |
| **Breast** |  |  |  |  |  |  |  |  |
| Do MH et al. (2003) | 1998-1999 | Hospital-based  (Hanyang and Soon-chunhyang University Hospital) | Cases: histologically confirmed incident cases  Controls: patients of the same hospital | 108 women | 121 women | <20.0  20~24  25~29  ≥30.0 | 1.00  1.12 (0.75-2.06)  0.98 (0.41-1.96)  1.98 (1.08-2.05) | Postmenopausal women  Adjusted for age |
| Han SH et al.  (2008) | 2001-2005 | Hospital-based  (Seoul National University and Asan Medical Center) | Cases: histologically  confirmed incident cases.  Controls: Cancer-free controls have DNA samples available | 635 women | 725 women | <25.0  25.0~29.9  ≥30.0 | 1.0  1.1 (0.82-1.39)  1.6 (0.84-3.03) | Postmenopausal women  Adjusted for age at  enrollment, education, age at first full term pregnancy or null parity, family history of breast cancer, and smoking status |
| Yoo KY et al. (1998) | 1994-1997 | Hospital-based  (Seoul National University Hospital) | Cases: histologically confirmed incident cases  Controls: patients of the same hospital | 280 women | 930 women | ≤20  20~22  22~24  24~26  26< | 1.00  1.26 (0.40-3.92)  1.80 (0.65-5.00)  2.68 (0.93-7.70)  3.00 (1.07-8.40) | Postmenopausal women  Adjusted for age and  education |
| **Uterine Corpus** | |  |  |  |  |  |  |  |
| Tong SY et al  (2009) | 1998-2006 | Tumor registry  databases of  Medical centers  in Korea | Cases; histologically  confirmed endometrioid  adenocarcinoma  Control;  Benign gynaecologic disease | 125  women | 302  women | ~22.9  23.0~24.9  ≥25 | 1.00  1.19 (0.62-2.29)  2.65 (1.44-4.89) | Adjusted for age |
|  |  |  |  |  |  | <25.0  25.0~29.9  ≥30.0 | 1.00  1.14(1.03-1.27)  1.25(1.04-1.50) | log dose- response method |
