# Supplementary material for: Population-Attributable Causes of Cancer in Korea: Obesity and Physical Inactivity
Source: PLoS One. 2014 Apr 10;9(4):e90871. doi: 10.1371/journal.pone.0090871 (PMC3982956; doi:10.1371/journal.pone.0090871)
Supplement: Table S2 — Studies included in the meta-analysis for physical inactivity. (DOCX) [file pone.0090871.s002.docx]

Table S2. Studies included in the meta-analysis physical inactivity

| Author (year) | Study period | Study subjects | | | | Category of  Physical inactivity | OR  (95% CI) | Confounding variables  considered |
| --- | --- | --- | --- | --- | --- | --- | --- | --- |
|  |  | Type and source | Definition | No. of  cases | No. of controls |  |  |  |
| **Colorectum** |  |  |  |  |  |  |  |  |
| Kim DH et al  (2002) | 1995-1996 | Hospital- based  (Seoul National University Hospital, National Medical Center, Korea Cancer Center Hospital) | Cases: histologically confirmed incident cases  Controls: patients of the same hospital | 138 men  97 women | 69 men  59 women | Sedentary  Moderate  Active | 1.00  1.01 (0.5-1.9)  1.29 (0.6-2.6) | Adjusted for age, sex, education level, job activity, hospital of admission,  Meat intake, fiber intake, and total Energy intake |
| Yun YH et al  (2008) | 1996 | National Health Insurance  Corporation (NHIC) | Cohort study  444,963 men | 1,076  751 |  | Low  Moderate-high | 1.00  0.98 (0.90-1.08) | Adjusted for age, dietary preference, LPA, smoking status, amount of alcohol drinking, body mass index,  employment and fasting blood sugar as appropriate |
| **Breast** |  |  |  |  |  |  |  |  |
| Do MH et al. (2003) | 1998-1999 | Hospital-based  (Hanyang and Soon-chunhyang University Hospital) | Cases: histologically confirmed incident cases  Controls: patients of the same hospital | 12  12 | 18  20 | 1-2  ≥3 | 1.00  1.11(0.35-3.49) | Pooled Estimation  Frequency of excise(per week) |
