# Supplementary material for: Population-Attributable Causes of Cancer in Korea: Obesity and Physical Inactivity
Source: PLoS One. 2014 Apr 10;9(4):e90871. doi: 10.1371/journal.pone.0090871 (PMC3982956; doi:10.1371/journal.pone.0090871)
Supplement: Table S3 — Estimated number of cancer incidence cases attributable to excess body weight in the Republic of Korea (using Caucasian cut-offs). (DOCX) [file pone.0090871.s003.docx]

Table S3. Estimated number of cancer incidence cases attributable to excess body weight in the Republic of Korea (using Caucasian cut-offs)

| Cancer site | Men | |  | Women | |  | Total | | |
| --- | --- | --- | --- | --- | --- | --- | --- | --- | --- |
|  | PAF(%) | Cases |  | PAF(%) | Cases |  | PAF(%) | Cases | PAF(%) |
| Colorectum | 2.53 | 378 |  | 0.03 | 3 |  | 1.54 | 381 | 1.42 |
| Pancreas | 1.95 | 46 |  | 10.16 | 193 |  | 5.61 | 239 | 5.64 |
| Kidney | 3.14 | 72 |  | 0.67 | 7 |  | 2.35 | 79 | 2.43 |
| Breast | - | - |  | 4.33 | 274 |  | 4.33 | 274 | 4.33 |
| Corpus uteri | - | - |  | 4.12 | 71 |  | 4.12 | 71 | 4.12 |
| Total | 0.51 | 496 |  | 0.60 | 548 |  | 0.56 | 1,044 |  |

* BMI categories (kg/m^2^) for Caucasians: reference group, <25.0; overweight, 25.0–29.9; obese, ≥30.0.
