# Supplementary material for: Population-Attributable Causes of Cancer in Korea: Obesity and Physical Inactivity
Source: PLoS One. 2014 Apr 10;9(4):e90871. doi: 10.1371/journal.pone.0090871 (PMC3982956; doi:10.1371/journal.pone.0090871)
Supplement: Table S4 — Distribution of age, obesity, smoking and drinking in Korean men. (DOCX) [file pone.0090871.s004.docx]

Table S4. Distribution of age, obesity, smoking and drinking in Korean men

|  | Age < 50 years |  |  |  |  |  | | Age ≥ 50 years |  |  |  |
| --- | --- | --- | --- | --- | --- | --- | --- | --- | --- | --- | --- |
| Obesity | Smoking | % within obesity category | Drinking | % within smoking category |  | Obesity | Smoking | | % within obesity category | Drinking | % within smoking category |
| BMI < 23 | Non-smokers | 18.3 | Non-drinkers | 36.5 |  | BMI < 23 | Non-smokers | | 15.8 | Non-drinkers | 47.5 |
|  |  |  | Drinkers | 63.5 |  |  |  | |  | Drinkers | 52.5 |
|  | Ever smokers | 17.1 | Non-drinkers | 24.3 |  |  | Ever smokers | | 22.3 | Non-drinkers | 37.6 |
|  |  |  | Drinkers | 75.7 |  |  |  | |  | Drinkers | 62.4 |
|  | Current smokers | 64.6 | Non-drinkers | 15.6 |  |  | Current smokers | | 62.0 | Non-drinkers | 25.5 |
|  |  |  | Drinkers | 84.4 |  |  |  | |  | Drinkers | 74.5 |
| 23≤ BMI < 25 | Non-smokers | 19.5 | Non-drinkers | 29.9 |  | 23≤ BMI < 25 | Non-smokers | | 20.7 | Non-drinkers | 40.1 |
|  |  |  | Drinkers | 70.1 |  |  |  | |  | Drinkers | 59.9 |
|  | Ever smokers | 19.1 | Non-drinkers | 20.8 |  |  | Ever smokers | | 27.1 | Non-drinkers | 31.3 |
|  |  |  | Drinkers | 79.2 |  |  |  | |  | Drinkers | 68.7 |
|  | Current smokers | 61.3 | Non-drinkers | 13.1 |  |  | Current smokers | | 52.3 | Non-drinkers | 22.6 |
|  |  |  | Drinkers | 86.9 |  |  |  | |  | Drinkers | 77.4 |
| 25≤ BMI < 30 | Non-smokers | 19.8 | Non-drinkers | 25.6 |  | 25≤ BMI < 30 | Non-smokers | | 23.4 | Non-drinkers | 37.3 |
|  |  |  | Drinkers | 74.4 |  |  |  | |  | Drinkers | 62.7 |
|  | Ever smokers | 19.1 | Non-drinkers | 19.6 |  |  | Ever smokers | | 29.0 | Non-drinkers | 31.1 |
|  |  |  | Drinkers | 80.4 |  |  |  | |  | Drinkers | 68.9 |
|  | Current smokers | 61.1 | Non-drinkers | 12.2 |  |  | Current smokers | | 47.6 | Non-drinkers | 22.2 |
|  |  |  | Drinkers | 87.8 |  |  |  | |  | Drinkers | 77.8 |
| BMI≥30 | Non-smokers | 18.7 | Non-drinkers | 24.7 |  | BMI≥30 | Non-smokers | | 26.4 | Non-drinkers | 38.4 |
|  |  |  | Drinkers | 75.3 |  |  |  | |  | Drinkers | 61.6 |
|  | Ever smokers | 16.4 | Non-drinkers | 22.7 |  |  | Ever smokers | | 28.1 | Non-drinkers | 36.7 |
|  |  |  | Drinkers | 77.3 |  |  |  | |  | Drinkers | 63.3 |
|  | Current smokers | 65.0 | Non-drinkers | 14.0 |  |  | Current smokers | | 45.5 | Non-drinkers | 20.5 |
|  |  |  | Drinkers | 86.0 |  |  |  | |  | Drinkers | 79.5 |
| 0.389^a^ |  | < .0001^b^ |  | <.0001 ^c^ |  |  |  | | < .0001 ^b^ |  | <.0001 ^c^ |

^a^ χ^2^ test on BMI and age; ^b^ χ^2^ test on BMI and smoking; ^c^ Mantel-Haenzel χ^2^ test on drinking and smoking stratified by BMI.
