# Supplementary material for: Population-Attributable Causes of Cancer in Korea: Obesity and Physical Inactivity
Source: PLoS One. 2014 Apr 10;9(4):e90871. doi: 10.1371/journal.pone.0090871 (PMC3982956; doi:10.1371/journal.pone.0090871)
Supplement: Table S5 — Distribution of age, obesity, smoking and drinking in Korean women. (DOCX) [file pone.0090871.s005.docx]

Table S5. Distribution of age, obesity, smoking and drinking in Korean women

|  | Age < 50 years |  |  |  |  |  | Age ≥ 50 years |  |  |  |
| --- | --- | --- | --- | --- | --- | --- | --- | --- | --- | --- |
| Obesity | Smoking | % smoking  within obesity category | Drinking | % drinking  within smoking category |  | Obesity | Smoking | % smoking  within obesity category | Drinking | % drinking  within smoking category |
| BMI < 23 | Non-smokers | 98.8 | Non-drinkers | 85.3 |  | BMI < 23 | Non-smokers | 81.6 | Non-drinkers | 89.6 |
|  |  |  | Drinkers | 14.7 |  |  |  |  | Drinkers | 10.4 |
|  | Ever smokers | 0.5 | Non-drinkers | 49.8 |  |  | Ever smokers | 5.3 | Non-drinkers | 72.8 |
|  |  |  | Drinkers | 50.2 |  |  |  |  | Drinkers | 27.2 |
|  | Current smokers | 0.7 | Non-drinkers | 40.6 |  |  | Current smokers | 13.0 | Non-drinkers | 66.2 |
|  |  |  | Drinkers | 59.4 |  |  |  |  | Drinkers | 33.8 |
| 23≤ BMI < 25 | Non-smokers | 98.1 | Non-drinkers | 85.7 |  | 23≤ BMI < 25 | Non-smokers | 87.3 | Non-drinkers | 89.8 |
|  |  |  | Drinkers | 14.3 |  |  |  |  | Drinkers | 10.2 |
|  | Ever smokers | 0.7 | Non-drinkers | 49.8 |  |  | Ever smokers | 4.5 | Non-drinkers | 76.6 |
|  |  |  | Drinkers | 50.2 |  |  |  |  | Drinkers | 23.4 |
|  | Current smokers | 1.2 | Non-drinkers | 43.7 |  |  | Current smokers | 8.2 | Non-drinkers | 68.6 |
|  |  |  | Drinkers | 56.3 |  |  |  |  | Drinkers | 31.4 |
| 25≤ BMI < 30 | Non-smokers | 97.5 | Non-drinkers | 85.3 |  | 25≤ BMI < 30 | Non-smokers | 88.3 | Non-drinkers | 88.9 |
|  |  |  | Drinkers | 14.7 |  |  |  |  | Drinkers | 11.1 |
|  | Ever smokers | 0.8 | Non-drinkers | 52.8 |  |  | Ever smokers | 4.9 | Non-drinkers | 75.4 |
|  |  |  | Drinkers | 47.2 |  |  |  |  | Drinkers | 24.6 |
|  | Current smokers | 1.7 | Non-drinkers | 42.0 |  |  | Current smokers | 6.8 | Non-drinkers | 70.2 |
|  |  |  | Drinkers | 58.0 |  |  |  |  | Drinkers | 29.8 |
| BMI≥30 | Non-smokers | 95.2 | Non-drinkers | 83.7 |  | BMI≥30 | Non-smokers | 87.6 | Non-drinkers | 88.1 |
|  |  |  | Drinkers | 16.3 |  |  |  |  | Drinkers | 11.9 |
|  | Ever smokers | 1.5 | Non-drinkers | 61.5 |  |  | Ever smokers | 4.8 | Non-drinkers | 82.0 |
|  |  |  | Drinkers | 38.5 |  |  |  |  | Drinkers | 18.0 |
|  | Current smokers | 3.3 | Non-drinkers | 49.4 |  |  | Current smokers | 7.6 | Non-drinkers | 75.8 |
|  |  |  | Drinkers | 50.6 |  |  |  |  | Drinkers | 24.2 |
| <.0001 ^a^ |  | <.0001 ^b^ |  | <.0001 ^c^ |  |  |  | <.0001 ^b^ |  | <.0001 ^c^ |

^a^ χ^2^ test on BMI and age; ^b^ χ^2^ test on BMI and smoking; ^c^ Mantel-Haenzel χ^2^ test on drinking and smoking stratified by BMI.
